# The H-Index as a Quantitative Indicator of the Relative Impact of Human Diseases

**DOI:** 10.1371/journal.pone.0019558

**Published:** 2011-05-19

**Authors:** K. Marie McIntyre, Iain Hawkes, Agnès Waret-Szkuta, Serge Morand, Matthew Baylis

**Affiliations:** 1 LUCINDA Group, Department of Epidemiology & Population Health, Institute of Infection & Global Health, University of Liverpool, Cheshire, United Kingdom; 2 Unité de recherche (UR) Animal et Gestion Intégrée des Risques (AGIRs), La Recherche Agronomique pour le Développement/Agricultural Research for Development (CIRAD), Montpellier, France; 3 Institut des Sciences de l'Evolution, Centre National de la Recherche Scientifique (CNRS), Institut de Recherche pour le Développement (IRD), Université Montpellier, Montpellier, France; Massey University, New Zealand

## Abstract

Assessment of the relative impact of diseases and pathogens is important for agencies and other organizations charged with providing disease surveillance, management and control. It also helps funders of disease-related research to identify the most important areas for investment. Decisions as to which pathogens or diseases to target are often made using complex risk assessment approaches; however, these usually involve evaluating a large number of hazards as it is rarely feasible to conduct an in-depth appraisal of each. Here we propose the use of the H-index (or Hirsch index) as an alternative rapid, repeatable and objective means of assessing pathogen impact. H-index scores for 1,414 human pathogens were obtained from the Institute for Scientific Information's Web of Science (WOS) in July/August 2010. Scores were compared for zoonotic/non-zoonotic, and emerging/non-emerging pathogens, and across taxonomic groups. H-indices for a subset of pathogens were compared with Disability Adjusted Life Year (DALY) estimates for the diseases they cause. H-indices ranged from 0 to 456, with a median of 11. Emerging pathogens had higher H-indices than non-emerging pathogens. Zoonotic pathogens tended to have higher H-indices than human-only pathogens, although the opposite was observed for viruses. There was a significant correlation between the DALY of a disease and the H-index of the pathogen(s) that cause it. Therefore, scientific interest, as measured by the H-index, appears to be a reflection of the true impact of pathogens. The H-index method can be utilized to set up an objective, repeatable and readily automated system for assessing pathogen or disease impact.

## Introduction

Assessment of the potential impact of diseases and pathogens is important for international, national and regional agencies and other organisations charged with providing disease surveillance and mitigation measures including implementation of disease management and control. It is also useful for funders of research so that they can identify the most important areas for investment. Decisions as to which specific pathogens or diseases to target are often made using risk assessment techniques as prioritisation tools. However, this usually involves evaluating a large number of hazards where it is not feasible to conduct an in-depth appraisal of all pathogens or diseases. Qualitative and semi-quantitative risk assessment approaches are specifically criticised either due to their potential subjectivity or the large amount of time and physical resources they use, creating results which may no longer be accurate by the time they are published. However, most quantitative methods require some input of expert opinion giving them a degree of subjectivity as well. In addition, it can be difficult to identify both parameters and estimates of parameter effects within the scientific literature, which can then be used within a quantitative model. All risk assessments are therefore biased in some way; either by the quality of the evidence utilised, time taken for its collection and therefore the timeliness of results or by the opinion of experts employed to make judgements on topics.

Here we propose the use of the H-index (or Hirsch index) to assess pathogen impact. This index is a bibliometric indicator obtained using certain bibliographic software packages such as the Institute for Scientific Information's Web of Science (WOS) [1]. For any group of keywords or phrases, it measures the number of published papers, *N*, that have been cited *N* or more times. This simple measure has been found to be a useful indicator of both the technical productivity and the apparent scientific impact of an individual within the scientific community [2], and is used within the recruitment process for scientists. It combines elements of the quantity of work undertaken (the number of publications, *N*) and the quality of work undertaken (the number of citations, also *N*). While the H-index is undeniably crude, it has the advantages that for any given search term it takes only minutes to obtain and is user-objective; it lends itself therefore to comparison of a large number of terms (people, pathogens) rather than in-depth comparison of a few.

This study aimed to examine the potential use of the H-index as an objective, time and resource efficient measure for the prioritisation of pathogens of humans [3]. Evaluation of the relative impact of pathogens indicated by H-indices with their true impact was undertaken by comparing the disability adjusted life year (DALY) estimates for a subset of diseases with the H-index score for the pathogens that cause them.

The pathogen database being utilised as the study population also contained information on the taxonomic division in which pathogens belonged (bacteria and rickettsia (hereafter defined as bacteria), fungi, helminths, protozoa, viruses and prions (hereafter defined as viruses)), and whether pathogens were considered emerging or zoonotic [3]. Differences between H-index scores were therefore examined for emerging pathogens compared to those not considered emerging, and for pathogens considered to be zoonotic (transferable from animals to humans) compared to human-only pathogens, both generally or stratified by taxonomic division.

## Methods

### List of pathogens of humans

A previously generated database of infectious organisms known to have pathogenic effects upon humans was utilised as the sample for investigation [3]. Due to difficulty in distinguishing between them within pathogen searches, European and Far eastern Tick–borne encephalitis (TBE) were combined in a search for TBE. In all, 1414 pathogens were therefore studied, of which 38.0% were bacteria, 21.7% were fungi, 20.3% were helminths, 4.7% were protozoa and 15.3% were viruses (table 1). 12.3% of pathogens were identified as emerging, and 61.2% were zoonotic. Emerging pathogens, as classified by [3], were defined as those that have appeared in a human population for the first time, or have occurred previously but are increasing in incidence or expanding into areas where they had not previously been reported, usually over the last 20 years. Zoonotic pathogens were defined as those naturally transmitted between vertebrate animals and man. Pathogens previously but no longer transmitted from animals, such as HIV, were not regarded as zoonotic.

**Table 1:** Number and percentage (n/%) of pathogens within taxonomic divisions and whether they are emerging or non-emerging and zoonotic or affect humans-only according to [3].

n/%	Total n = 1414	Emerging	Not emerging	Zoonotic	Human-only
Group		174/12.3	1240/87.7	866/61.2	548/38.8
Bacteria or rickettsia	538/38.0	53/9.9	485/90.1	269/50.0	269/50.0
Fungi	307/21.7	16/5.2	291/94.8	116/37.8	191/62.2
Helminths	287/20.3	10/3.5	277/96.5	274/95.5	13/4.5
Protozoa	66/4.7	19/28.8	47/71.2	43/65.2	23/34.8
Viruses or prions	216/15.3	76/35.2	140/64.8	164/75.9	52/24.1

### Literature searches

#### Identification of the H-index score of pathogens

H-index scores were obtained for all pathogen names as given by [3] using phrase searches enclosed using “” within WOS [1], although several spelling alterations were needed. All searches were for 1900 to 2009, inclusive, and they were undertaken between July and August 2010. Searches for viruses were more complex, however, because of the existence of synonyms and acronyms. Synonyms and acronyms were obtained from the NCBI taxonomy website (http://www.ncbi.nlm.nih.gov/Taxonomy/taxonomyhome.html) or http://www.ictvdb.org/Ictv/index.htm and included as additional search terms. However, it was observed that some acronyms were used for more than one virus, or occurred in a non-viral context. All searches for viruses therefore also included the term ‘virus’ and excluded any other entities (viral or non-viral) which shared the acronym. The details for pathogens with the top 20 H-index scores are presented in table 2.

**Table 2:** Infectious organisms pathogenic to humans with the top 20 H-index scores following searches of the literature using WOS [1].

Pathogen Name	Group	H-index score
*Saccharomyces cerevisiae*	Fungi	456
Human Immunodeficiency Virus 1	Viruses	349
Hepatitis A virus	Viruses	317
Hepatitis C virus	Viruses	276
*Staphylococcus aureus*	Bacteria	253
Hepatitis B virus	Viruses	236
*Helicobacter pylori*	Bacteria	227
Human papillomavirus	Viruses	227
*Pseudomonas aeruginosa*	Bacteria	225
*Salmonella* Typhimurium	Bacteria	225
*Mycobacterium tuberculosis*	Bacteria	224
*Bacillus subtilis*	Bacteria	206
*Escherichia coli*	Bacteria	206
*Plasmodium falciparum*	Protozoa	199
*Listeria monocytogenes*	Bacteria	198
*Streptococcus pneumoniae*	Bacteria	186
*Candida albicans*	Fungi	171
Vesicular stomatitis virus	Viruses	169
*Leishmania major*	Protozoa	152
Human Herpesvirus 4	Viruses	147

### Comparison of H-index scores and DALY estimations

DALYs [4] were developed by the World Health Organization, and are suggested to be the best measure of the true burden of disease. They combine morbidity and mortality within a single metric by including equivalent years of ‘healthy’ life lost by virtue of being in states of poor health or disability, with an estimation of the potential years of life lost due to premature death. Japanese life expectancy statistics are used as the standard for measuring premature death, and one DALY is equivalent to one year of healthy life lost. The estimations used within the study were taken from the most recent Global Burden of Disease report [5]. As DALY estimations are only calculated for well known and high impact clinical diseases, comparison was restricted to a short-list of the pathogens for which H-index scores were calculated (n = 27). As DALY estimations are of clinical diseases rather than for the effects of specific pathogens, clinical ailments caused by pathogens were established, with several pathogens in some cases causing a single clinical disease. Hence, further bibliographic searches using WOS were undertaken to re-obtain H-index scores for a combination of pathogen names, synonyms, acronyms and disease name. Such searches identified a specific problem for AIDS/HIV, with the number of papers identified above the maximum WOS search threshold of 100 000; search terms were removed until the total number was just under this threshold and an estimate of the H-index score could therefore be calculated. To clarify that H-index scores are comparable across different bibliographic indexes which search various literature sources over differing temporal periods, H-index scores for a sub-sample of pathogen names were also derived from searches using SCOPUS [6] and Google Scholar [7] software.

### Statistical analyses

For analyses, parametric methods were used where possible, or non-parametric approaches were employed if necessary. The variance structures of H-index scores for grouped emerging versus non-emerging, and zoonotic versus non-zoonotic pathogens and for those stratified by taxonomic division were examined using Levene's tests. If the two pairs of data had different variances, then they were either log_10_+1 transformed or if this did not normalise the data then non-parametric statistical methods were thereafter adopted. Differences between H-index scores for emerging versus non-emerging pathogens, and zoonotic versus non-zoonotic pathogens, were examined using either a 2-sample T-test or Mann-Whitney U test (*P*<0.05).

To examine whether the H-index scores of taxonomic divisions differed from each other, a Kruskal-Wallis test was used, after an Anderson–Darling normality test had first been employed to examine the distribution of the data. Multiple comparison testing to identify differences between the H-index scores of taxonomic divisions was also undertaken [8], [9].

Examination of the suitability of using the H-index score of pathogens as an indicator of their impact upon humans was undertaken by comparison with DALY value estimations previously published by the World Health Organisation [5]. In this case, the DALY estimates for a number of diseases were compared with the combined H-index for the most important pathogens that cause the diseases as well as the disease name. Anderson–Darling normality tests were initially used to examine whether the DALY and H-index score data were normally distributed; the data were log-transformed prior to analysis by Pearson product-moment correlation (*P*>0.05). Comparison of H-index scores derived from other literature sources was undertaken using Spearman Rank correlation (*P*>0.05) as the data were non-normally distributed even after transformation.

## Results

**Figure 1:**
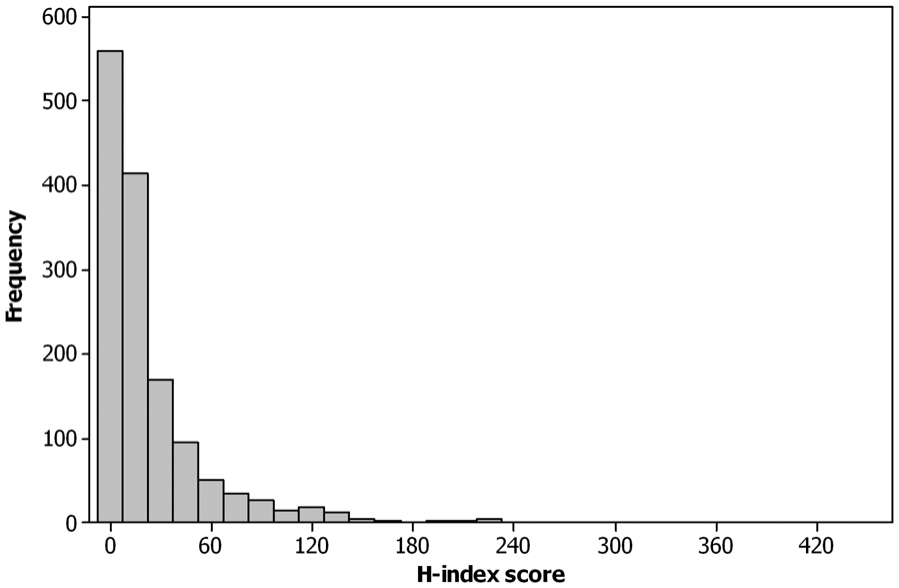
Frequency histogram of the H-index scores obtained using phrase searches within Web of Science [1] for the names of pathogens of humans according to [3].

The H-index scores for pathogens from the database of infectious organisms [3] were highly over-dispersed (minimum value = 0, maximum value = 456, mean value = 25, median value = 11, standard deviation = 38; figure 1, table 2) with most pathogens producing relatively low scores. Those pathogens with the highest scores were examples of the following: person-to-person transmitted viruses (Hepatitis A, B or C virus, Human Herpesvirus 4, Human Immunodeficiency Virus 1, Human papillomavirus) or bacteria (*Helicobacter pylori*, *Mycobacterium tuberculosis*), agents causing opportunistic oral and genital infection (*Candida albicans*), bacteria causing multiple clinical symptoms (*Staphylococcus aureus*, *Pseudomonas aeruginosa*, *Salmonella* Typhimurium, *Streptococcus pneumonia*), food-borne bacterial pathogens (*Escherichia coli*, *Listeria monocytogenes*), model organisms for laboratory studies (*Bacillus subtilis*, Vesicular stomatitis virus), major tropical illnesses (*Plasmodium falciparum*, *Leishmania major*) or yeast (*Saccharomyces cerevisiae*); an occasional opportunistic infection but mainly used within the brewing and baking industries.

**Figure 2:**
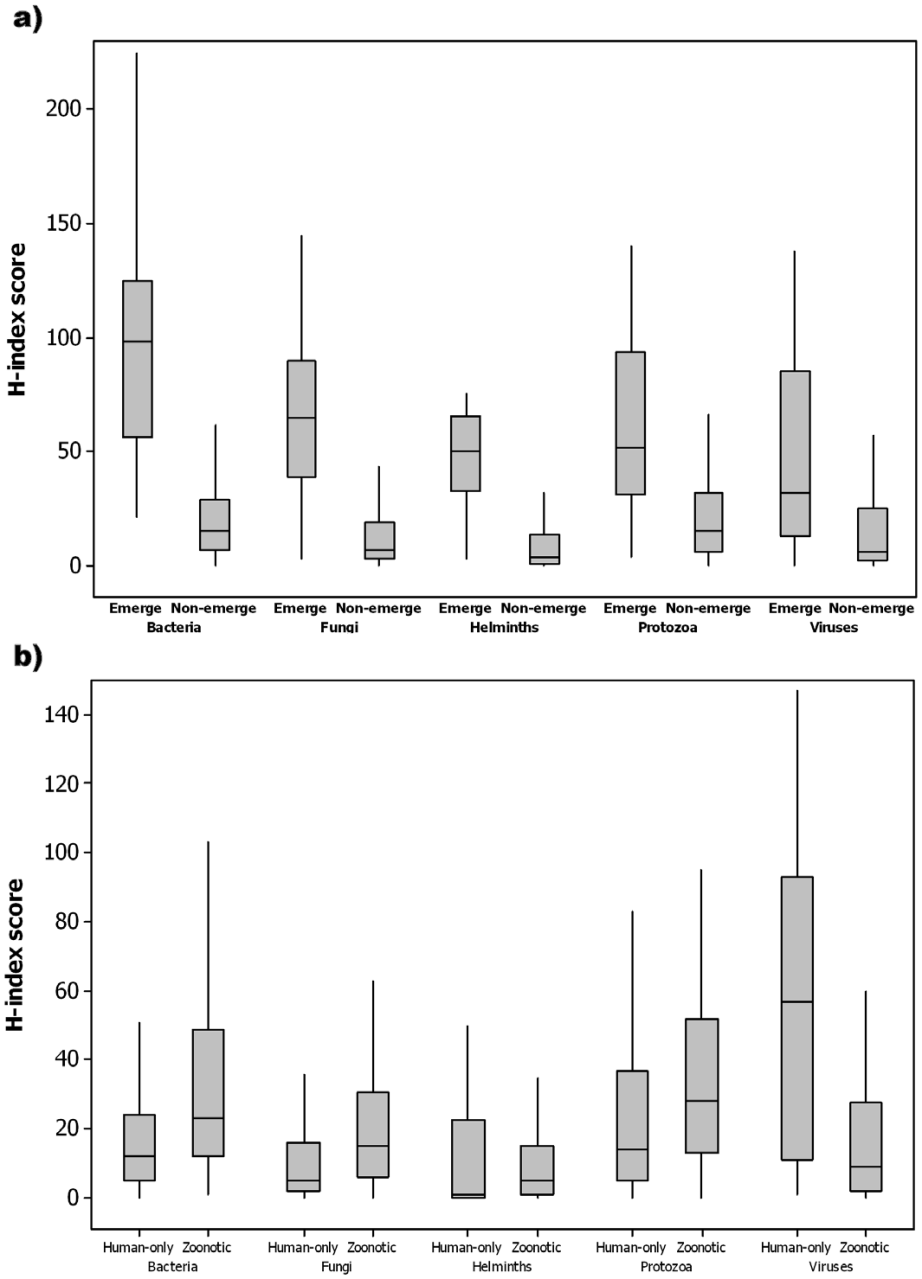
H-index scores by taxonomic division for (a) emerging and non-emerging or (b) zoonotic and human-only pathogens of humans, both according to [3].

Emerging pathogens had significantly higher H-index scores than non-emerging pathogens (figure 2a; median values: 55.0 versus 10.0, respectively, Mann-Whitney U test, *P*<0.001). For zoonotic versus human-only pathogens, there was no significant difference in their mean scores (figure 2b; mean values: 25.5 versus 23.8, respectively, 2-sample T-test, *P* = 0.425). Once the scores had been stratified by taxonomic division, emerging bacteria, fungi, helminths, protozoa and viruses all had significantly higher H-index scores than non-emerging (*P*<0.001, table 3, figure 2a). In addition, zoonotic pathogens within the bacteria and fungi divisions had significantly higher H-index scores (*P*<0.001, table 4, figure 2b) than human-only, but human-only viruses and prions had significantly higher scores than zoonotic viruses (*P*<0.001, table 4, figure 2b).

**Table 3:** The results of tests of equal variances, 2-sample T-tests or Mann-Whitney U tests on (log_10_+1 transformed) H-index scores for emerging and non-emerging pathogens by taxonomic division.

Taxonomic division	H-index score test of equal variances	H-index score (Log_10_+1) test of equal variances	T-test	Mann-Whitney U test
Bacteria	-	P<0.001	-	P<0.001
Fungi	-	P = 0.024	-	P<0.001
Helminth	-	P = 0.021	-	P<0.001
Protozoa	-	P = 0.143	P<0.001	-
Virus	-	P = 0.087	P<0.001	-

**Table 4:** The results of tests of equal variances, 2-sample T-tests or Mann-Whitney U tests on (log_10_+1 transformed) H-index scores for zoonotic and human-only pathogens by taxonomic division.

Taxonomic division	H-index score test of equal variances	H-index score (Log10+1) test of equal variances	T-test	Mann-Whitney U test
Bacteria	P<0.001	P = 0.706	P<0.001	-
Fungi	P = 0.185	P = 0.106	P<0.001	-
Helminth	P = 0.572	-	P = 0.851	-
Protozoa	P = 0.597	-	P = 0.322	-
Virus	P<0.001	P<0.001	-	P<0.001

There were significant differences between the H-index scores of some taxonomic groups (Kruskal-Wallis test, *P*<0.001), with those of bacteria significantly higher than those of the fungi (*P*<0.001), and helminth groups (*P*<0.001), and the scores of the protozoa group higher than those of fungi (*P* = 0.002).

**Figure 3:**
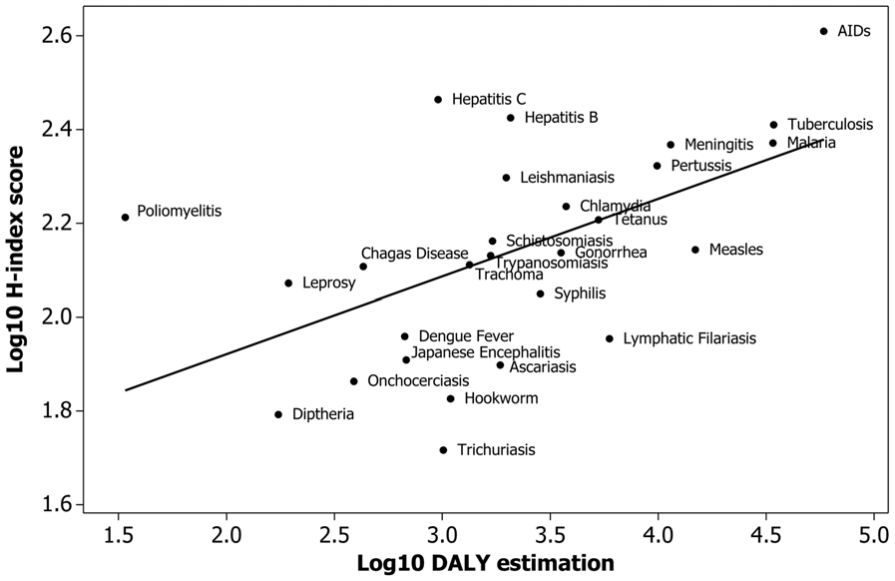
Relationship between burden of disease in log_10_-transformed Disability-adjusted-life-years (DALYs) and H-index scores.

Both the H-index score and DALY estimation data were non-normally distributed (*P* = 0.029 and *P*<0.010). After log_10_+1 transformation they were significantly positively correlated (figure 3, Pearson correlation coefficient = 0.546, *P* = 0.003).

H-index scores calculated using different bibliographic indexes were significantly positively correlated (WOS and SCOPUS H-index comparison, *P*<0.001; WOS and Google Scholar H-index comparison, *P*<0.001).

## Discussion

This study aimed to examine the use of the H-index as a tool for assessing the relative impact of pathogens. There are a number of weaknesses to this approach: the results are susceptible to a lag in time-to-publication, with newly emerging pathogens likely to be under-represented and thus have low H-index scores; the method needs some manual oversight, as false positives can occur for instance when pathogens are used as model organisms; biases in results may happen because of trends in interest in specific pathogens, diseases or research fields (as a result of regional publication biases or the Matthew effect; ‘the rich get richer and the poor get poorer’); in addition, results will need updating to allow for the inclusion of new research material. The literature searching method also doesn't account for the quality of publications in which pathogen names appear, or the typical number of citations within different fields. In addition, all bibliographic software packages incorporate newly published literature into their databases at different rates and the literature sources included are not identical in each [10]. Most importantly, the H-index method does not really measure ‘impact’; it measures scientific interest in a pathogen or disease.

However, there are also many advantages to the use of the H-index method. It can be rapidly obtained (one person obtained the 1414 H-indices in two weeks) and has the potential to be automated and repeated regularly. It is user-objective, and provides an easily understood quantitative measure. The scores reflect the wider scientific interest that would be expected to follow from a pathogen being either zoonotic or emerging. Most importantly, for a small subset of diseases for which their true impact has been estimated in terms of DALYS, there is a significant correlation with H-index scores. Scientific interest, as measured by the H-index appears to be, therefore, a reasonable reflection of the true impact of pathogens. In view of this relationship, outlying points below or above the line are diseases which have, respectively, relatively low or high scientific interest considering their true impact (as defined by their DALY estimation). Intriguingly, the strongest negative outliers are lymphatic filariasis, ascariasis, hookworm and trichuriasis; all four of which are nematode infections prevalent in developing countries. In other words, the H-index may provide a visual representation of the neglect of certain tropical diseases within the literature, either because of a lack of funding for research or from lack of publication of research findings. By contrast, the strongest positive outliers are poliomyelitis, hepatitis B and C and AIDS; three out of four of which are significant health problems in the developed world.

To conclude, this work suggests that scientific interest, indicated by H-index scores, is a reasonable reflection of the true impact of pathogens. Further research may show that a rapid increase in an H-index score can perhaps be used as an indicator of disease or pathogen emergence. A full list of H-index scores can be obtained from the authors.
